# The Role of Tapered Light-Diffusing Fibers in Plasmonic Sensor Configurations

**DOI:** 10.3390/s21196333

**Published:** 2021-09-22

**Authors:** Nunzio Cennamo, Francesco Arcadio, Luigi Zeni, Ester Catalano, Domenico Del Prete, Gionatan Buonanno, Aldo Minardo

**Affiliations:** Department of Engineering, University of Campania Luigi Vanvitelli, Via Roma 29, 81031 Aversa, Italy; francesco.arcadio@unicampania.it (F.A.); luigi.zeni@unicampania.it (L.Z.); ester.catalano@unicampania.it (E.C.); domenico.delprete1@studenti.unicampania.it (D.D.P.); gionatan.buonanno@studenti.unicampania.it (G.B.); aldo.minardo@unicampania.it (A.M.)

**Keywords:** surface plasmon resonance (SPR), optical fiber sensors, tapered light-diffusing fibers, light-diffusing fibers, modal filter

## Abstract

In this work, we experimentally analyzed the effect of tapering in light-diffusing optical fibers (LDFs) when employed as surface plasmon resonance (SPR)-based sensors. Although tapering is commonly adopted to enhance the performance of plasmonic optical fiber sensors, we have demonstrated that in the case of plasmonic sensors based on LDFs, the tapering produces a significant worsening of the bulk sensitivity (roughly 60% in the worst case), against a slight decrease in the full width at half maximum (FWHM) of the SPR spectra. Furthermore, we have demonstrated that these aspects become more pronounced when the taper ratio increases. Secondly, we have established that a possible alternative exists in using the tapered LDF as a modal filter after the sensible region. In such a case, we have determined that a good trade-off between the loss in sensitivity and the FWHM decrease could be reached.

## 1. Introduction

In recent years, surface plasmon resonance (SPR) has been demonstrated to be a very sensitive technique for the detection of small refractive index (RI) variations at the boundary between a thin metal layer (e.g., gold) and a dielectric medium (e.g., receptor layers).

In the literature, several sensor configurations that take advantage of the SPR phenomenon have been reported [1,2,3]. Furthermore, when using an optical fiber as the SPR medium, additional advantages, such as immunity to electromagnetic interference, remote sensing capabilities, a light weight, etc., can be obtained [4,5,6,7,8,9]. In this case, both silica and polymeric optical fibers (POF) can be used to develop groundbreaking sensors in several application fields [10,11,12,13].

In general, several solutions and configurations can be adopted to improve the performance of SPR sensors based on optical fibers, for instance, by using a bimetallic layer, modal filters, a doped fiber core, etc. [14,15,16,17]. A commonly used approach is tapering the sensing region, which usually enhances the performance [18]. The advantages of plasmonic sensors based on tapered optical fibers have been extensively explored in the literature, both from a theoretical and an experimental point of view [18,19,20,21,22]. Along this line, Cennamo et al. have recently presented an experimental analysis of SPR sensors based on tapered plastic optical fibers, confirming the theoretical predictions [23]. Furthermore, SPR sensors based on tapered optical fibers have been exploited in several application fields, such as water pollutant detection [24,25], magnetic field sensing [26], and many others [27,28,29].

Different kinds of light-diffusing fibers (LDFs) have recently shown their potentiality as plasmonic sensors [30,31,32]. This class of optical fibers is characterized by the fact that light is scattered out of the fiber all along its length due to light-scattering centers in its core. This characteristic allows for an easier fabrication procedure, since only a metal deposition step is required to build the SPR sensor [30].

In this work, we explored the use of tapered silica LDFs in plasmonic sensors configurations. As the first step, we conducted an experimental study on three different SPR-LDF sensor configurations, characterized by taper ratios of 1.3, 2.3, and 4.6, respectively. We then compared the performance parameters relative to an SPR sensor based on a straight (untapered) silica LDF and a plastic LDF. Finally, we reported the use of the tapered silica LDF as a modal filter, in similarity with the study reported in Ref. [15]. In all cases, several water–glycerin mixtures were used to test the proposed sensor configurations in terms of bulk sensitivity and FWHM.

This work aimed to demonstrate the effect the tapering process produced on a silica LDF plasmonic sensor. The choice of the used parameters was fixed at typical values compatible with the machine used, to compare the several tested sensor configurations.

## 2. Fabrication Process and Experimental Setup

### 2.1. Tapered LDF Probes

The silica LDF (Fibrance^®^ by Corning^®^, New York, NY, USA) used in our experiments was composed of a silica core with a diameter of 170 μm, and a polymeric cladding with a diameter of roughly 230 μm. The chosen fiber was a diffusive fiber with a plurality of helical voids randomly distributed into the core. The presence of the voids impacted the guided light, as it was scattered radially outward from the core. Clearly, the number of voids in each section and their dimensions caused a different amount of emitted light. In addition, the smaller pitches scattered more light than larger pitches, while their diameters ranged in size from 50 nm to 500 nm. Thus, the propagation light scattered independently of the wavelength of the used light source.

The tapered silica LDFs were fabricated using a GPX 3800 Glass Processing System (Vytran LLC, Morganville, NJ, USA). The tapers were created by heating the fiber to its softening point, and then pulling the ends apart to reduce its diameter. The GPX 3800 allowed us to precisely control the geometry of the taper, by regulating the filament power, the pull speed, and the fiber tension.

In this work, different tapers were utilized in order to characterize their roles in the plasmonic sensor system. More specifically, three different values of taper ratios were employed in our tests, i.e., 1.3, 2.3, and 4.6. In all cases, the total length of the fabricated taper was 3 cm, composed of a 1 cm long downtaper, a 1 cm-long waist, and a 1 cm long uptaper.

### 2.2. SPR Sensors Based on Straight LDFs and Tapered LDFs

In order to realize the SPR sensors based on straight (untapered) and tapered silica LDFs, a unique fabrication step was carried out, which consisted of depositing a thin gold nanofilm around the circumference of the sensible area (about 3 cm) [30]. This process was carried out through a sputter coater machine (Bal-Tec SDC 500, Schalksmühle, Germany). In order to metalize the whole fiber circumference along the sensible region, the deposition process was repeated twice, one for each side of the fiber. Each deposition step was performed for 105 s at a pressure of 0.05 mbar and a current of 60 mA, resulting in a gold layer thickness of roughly 60 nm, in a similar way to [30].

### 2.3. Equipment

The experimental setup was composed of a white light source and a spectrometer. The white light source (model HL-2000-LL, produced by Ocean Optics, Dunedin, FL, USA) emitted in a spectral range between 360 nm and 1700 nm, whereas the spectrometer (model FLAME-S-VIS-NIR-ES, produced by Ocean Optics, Dunedin, FL, USA) showed a detection range between 350 nm and 1023 nm, with a spectral resolution of 1.4 nm (FWHM).

The spectrometer was connected to a computer with specific software that managed and processed raw data [30].

To test the realized plasmonic sensor configurations based on the LDFs, different water–glycerin solutions were prepared, whose refractive indexes had been characterized by an Abbe refractometer (RMI, Exacta Optech, Munich, Germany).

### 2.4. Experimental Sensor Configurations

**Figure 1:**
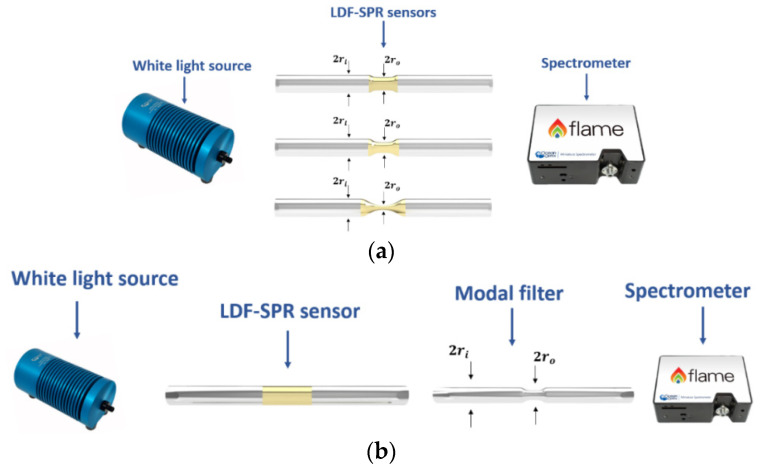
(**a**) Experimental tapered LDF-SPR sensors with three different taper ratios: 1.3, 2.3, and 4.6; (**b**) experimental SPR-LDF sensor configuration implementing a tapered LDF as a modal filter.

The experimental configurations comprised a white light source illuminating the plasmonic fiber sensors connected to a spectrometer (see Figure 1) [30]. The same equipment was used in the experimental analysis of all the plasmonic sensor configurations here reported.

Figure 1a demonstrates, in more detail, the first experimental configuration based on SPR-LDF sensors with a tapered sensing region. This kind of configuration was tested with three different taper ratios (1.3, 2.3, and 4.6), with the taper ratios being defined as the ratio between the diameter of the LDF (2 × r_i_) and the diameter of the taper in its waist region (2 × r_o_).

The second experimental configuration, shown in Figure 1b, was based on an SPR sensor followed by a modal filter, similar to the study reported in Ref. [15]. However, in this analysis, the plasmonic sensor was an SPR-LDF sensor, whereas the tapered LDF was used as a modal filter. In that case, the sensing region was composed of a piece of gold-coated, untapered LDF, with a length of 2 cm.

Before illustrating the results of our comparative analysis, let us recall the bulk sensitivity, the main parameter of interest for this kind of sensor. Sensitivity (*S*) can be defined as the shift in resonance wavelength per unit change of refractive index [33],

(1) $$S\;\left(n_{s}\right)={\left[\frac{\delta \lambda_{SPR}}{\delta n_{sm}}\right]}_{n_{s}}\;\left[\frac{nm}{RIU}\right]$$

where $\delta n_{sm}$ is the variation of the refractive index of the sensing medium giving rise to a resonance wavelength shift equal to $\delta \lambda_{SPR}$.

## 3. Experimental Results and Discussion

### 3.1. SPR Sensors Based on Tapered LDFs

The first experimental analysis regarded the comparison of three different plasmonic configurations based on tapered LDFs, with three different taper ratios (1.3, 2.3, and 4.6).

In this section, the results were obtained exploiting the experimental setup reported in Figure 1a.

**Figure 2:**
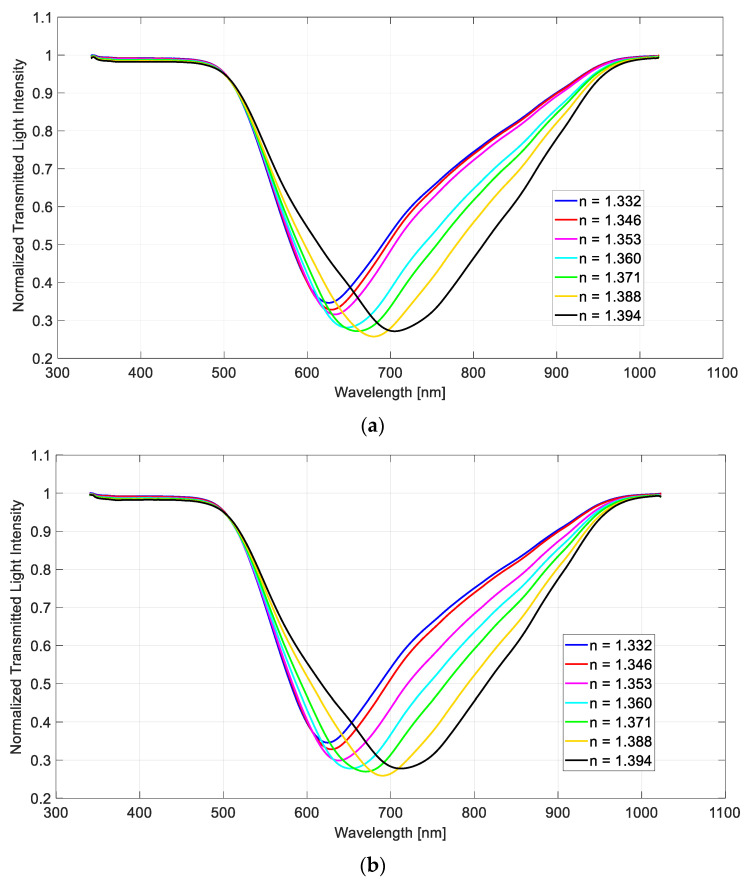

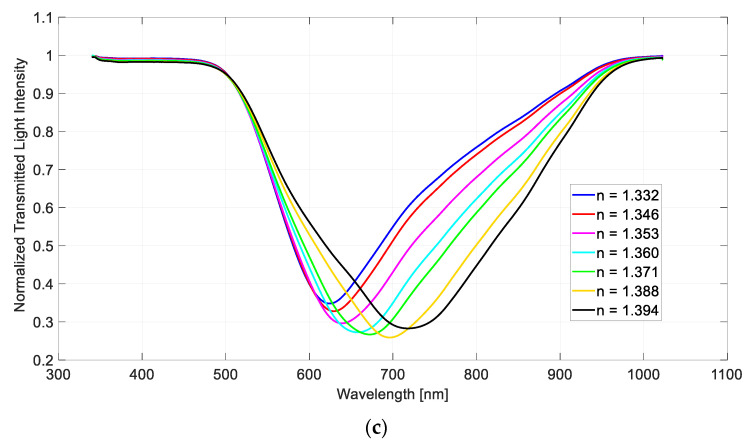
SPR transmitted spectra, obtained by normalization to the spectrum acquired with air as the surrounding medium, for different refractive index values. Configuration with a taper ratio equal to (**a**) 1.3, (**b**) 2.3, and (**c**) 4.6.

Figure 2 shows the transmission spectra at different taper ratios and external refractive indexes (*n*), as obtained by normalizing each spectrum to the one acquired with air as the surrounding medium, where the SPR resonance condition was not satisfied. In all three configurations, an increment of the resonance wavelength was observed when the refractive index of the surrounding medium grew. Figure 2 demonstrates that the significant differences of the experimental spectra were produced for high refractive indices values of the medium (higher than 1.353 RIU).

**Figure 3:**
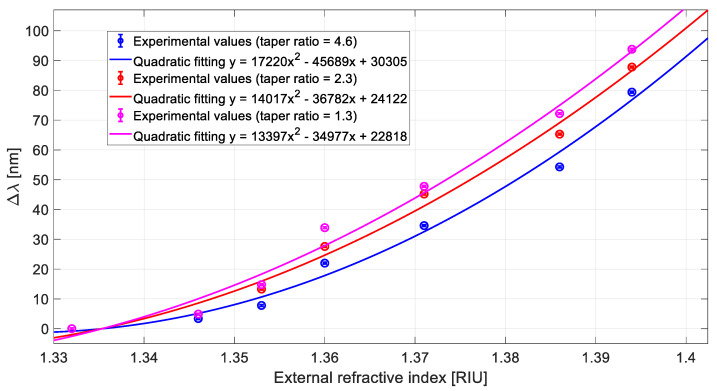
Variation in SPR wavelength (Δλ) versus external refractive index and quadratic fitting of the experimental data for three different configurations based on tapered LDFs.

This aspect can also be observed in Figure 3, where the experimental variation in resonance wavelength (Δλ), calculated with respect to water (*n* = 1.332), has been reported as a function of the external refractive index, for the three analysed sensor configurations. In Figure 3, each experimental value is the average of five consecutive measurements, while the error bars represent the highest measured standard deviation (equal to 0.2 nm). The quadratic fitting of the experimental results is reported as well.

**Figure 4:**
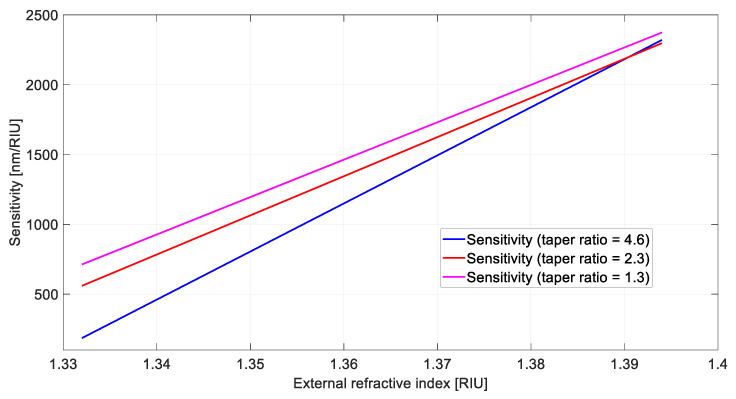
Sensitivity as a function of the refractive index for the configurations based on a tapered LDF.

Using Equation (1) and the quadratic fitting equations reported in Figure 3, we can approximate the bulk sensitivities as the first derivative of the quadratic functions, as shown in Figure 4, where it is evident that the sensitivity decreases as the taper ratio increases in the considered refractive index range.

Table 1 compares the sensitivity and the FWHM values of the sensor configurations based on a tapered silica LDF (with different taper ratios) and an untapered silica LDF [30], at a fixed refractive index equal to 1.353. The same table also reports the above-mentioned parameters for an LDF based on a POF without a taper [32]. The comparison demonstrates how tapering the LDF along the sensible region reduced the sensitivity in respect to the straight LDF, whereas the FWHM slightly decreased. This could be related to the way in which light propagates in silica LDFs. As already underlined, the silica LDF included in its core multiple centers of scattering that excited higher-order modes. Consequently, the SPR condition was easily satisfied, since a very large number of angles of incidence at the gold surface, capable of exciting plasmons, were obtained [30]. Therefore, the introduction of a tapered sensing region did not cause a variation in the resonance conditions in terms of useful SPR angles, which was the mechanism enhancing the SPR sensitivity when tapers were realized on a conventional optical fiber [18]. Rather, in our LDF, the only consequence of tapering, in terms of SPR excitation efficiency, was a reduction in the fiber diameter, which worsened the sensitivity while improving the capability to read the SPR minimum (the FWHM decreased), as already shown in SPR sensors based on POFs with different diameters [34].

### 3.2. SPR Sensor Based on LDFs and Modal Filter Based on Tapered LDF Probes

The second experimental configuration involved the use of a tapered silica LDF probe only as a modal filter. The use of modal filters has already been explored in the literature with different configurations [15,32]. In particular, the modal filter could be inserted either before (light source side) or after (spectrometer side) the sensor system. The second configuration (spectrometer side) was preferred because it led to major improvements in the capability to read the SPR minimum (decrease in the FWHM) [15,32].

For this reason, a modal filter consisting of a tapered silica LDF placed on the spectrometer side was implemented in our tests. The results were obtained in this section by utilizing the experimental setup reported in Figure 1b.

**Figure 5:**
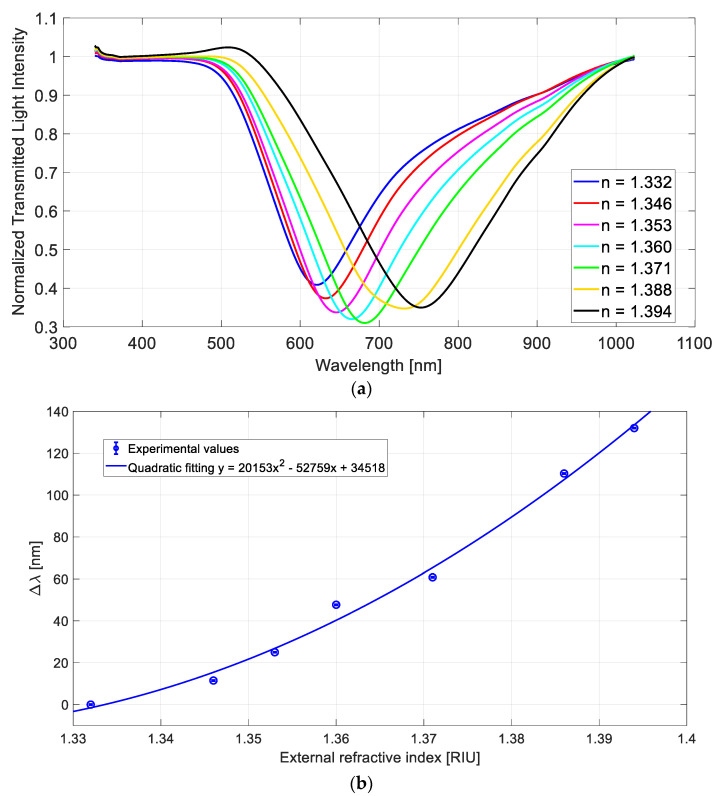
SPR-LDF configuration with a tapered silica LDF probe as a modal filter on the spectrometer side: (**a**) normalized SPR transmission spectra for different refractive index values; (**b**) variation in SPR wavelength (Δλ) versus external refractive index with the quadratic fitting of the experimental data and the error bars.

Figure 5a demonstrates the SPR transmission spectra using the tapered silica LDF probe as a modal filter, whereas Figure 5b demonstrates the variation in resonance wavelength (Δλ), calculated in respect to water (*n* = 1.332), with the quadratic fitting of the experimental values and the error bars.

### 3.3. Discussion

**Figure 6:**
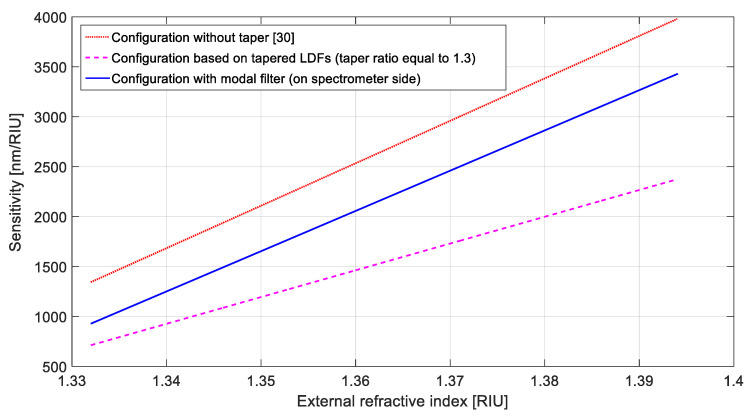
Sensitivity as a function of the refractive index for all the configurations based on silica LDF without a taper (red dots), silica LDF with a taper ratio equal to 1.3 (magenta dashes), and with a silica LDF as a modal filter on the spectrometer side (blue line).

In this section, we compare different plasmonic LDF-based sensor configurations. In particular, the sensitivity of the LDF-SPR sensor reported in [30] has been compared with the results here reported, obtained by utilizing the experimental configurations illustrated in Figure 1a (Section 3.1) and Figure 1b (Section 3.2). As such, Figure 6 reports the sensitivity to external refractive index changes of the SPR sensor configurations based on the untapered silica LDF [30], the tapered silica LDF (with a taper ratio equal to 1.3, the best configuration reported in Section 3.1), and the untapered silica LDFs with a tapered silica LDF employed as a modal filter (reported in Section 3.2).

It was clear that the modal filter led to an intermediate result in terms of sensitivity, since it ensured a higher sensitivity (in all the considered refractive index ranges) in respect to the best tapered configuration (Section 3.1).

Table 2 more generally demonstrates a comparative analysis of the LDF-based sensor configurations. As can be seen, the configuration based on a modal filter here presented could be a good trade-off between sensitivity and FWHM in respect to other sensor configurations [30,32]. In fact, the loss in sensitivity was reasonable (≈−19%), whereas the FWHM decreased by ≈ 4%. The FWHM reduction was extremely interesting because it could improve the capability to read the SPR minimum, making this kind of sensor simple to use in several potential application fields, exploiting friendly software interfaces. For example, by coupling these plasmonic probes with specific bio-receptors, sensitive biosensors could be easily integrable in a portable, simple-to-use, small-size, and low-cost device for point-of-care testing in different clinical applications.

As demonstrated in Table 2, in respect to the LDF-POF-based sensor [32], both configurations based on silica LDFs showed an overall improvement in the performance parameters.

## 4. Conclusions

We have analyzed a tapered silica LDF’s performance when it is employed in a plasmonic sensor system. At first, we determined that the tapering process, commonly used to enhance optical performances, led to a significant worsening in our study case. This aspect was related to the particular structure of the silica LDF. Moreover, we have shown that a good trade-off could be achieved using the tapered silica LDF not in the sensible region, but as a filter for the higher-order modes propagating inside the silica LDF, leading to an acceptable loss in sensitivity in favour of a reduction in the FWHM, by improving the capability to read the SPR minimum.

## Figures and Tables

**Table 1:** Comparison between different LDFs configurations, at a fixed refractive index equal to 1.353.

Plasmonic Sensor Configuration	Refractive Index (*n_s_*)	Sensitivity [nm/RIU]	FWHM [nm]	Reference
LDF in silica without taper	1.353	2200	173.6	[30]
LDF in silica with taper ratio of 1.3	1.353	1300	168.6	This work
LDF in silica with taper ratio of 2.3	1.353	1150	165.3	This work
LDF in silica with taper ratio of 4.6	1.353	910	157.6	This work
LDF-POF	1.353	1550	202.7	[32]

**Table 2:** Comparison of performances parameters relative to several configurations based on silica LDFs (without taper and with taper as a modal filter) and plastic LDFs (with a modal filter) at a fixed refractive index equal to 1.353.

Plasmonic Sensor Configuration	Refractive Index (*n*)	Sensitivity [nm/RIU]	FWHM [nm]	Reference
LDF in silica without taper	1.353	2200	173.6	[30]
LDF-POF sensor with modal filter on spectrometer side	1.353	1390	182.7	[32]
LDF in silica with modal filter on spectrometer side realized by a taper	1.353	1780	166.2	This work

## Data Availability

The data is available on reasonable request from the corresponding author.
